# Ectopic Expression of *AhGLK1b* (GOLDEN2-like Transcription Factor) in *Arabidopsis* Confers Dual Resistance to Fungal and Bacterial Pathogens

**DOI:** 10.3390/genes11030343

**Published:** 2020-03-24

**Authors:** Niaz Ali, Hua Chen, Chong Zhang, Shahid Ali Khan, Mamadou Gandeka, Dongyang Xie, Weijian Zhuang

**Affiliations:** 1 Oil Crops Research Institute, Fujian Provincial Key Laboratory of Plant Molecular and Cell Biology, Fujian Agriculture and Forestry University, Fuzhou, Fujian 350002, China; niazaliadil@yahoo.com (N.A.); hchen2013@fafu.edu.cn (H.C.); zhangchong20022008@163.com (C.Z.); shahidalikhan053@gmail.com (S.A.K.); mgandeka@yahoo.fr (M.G.); zhuwxie@126.com (D.X.); 2 College of Crop Science, Fujian Agriculture and Forestry University, Fuzhou, Fujian 350002, China; 3 College of Plant Protection, Fujian Agriculture and Forestry University, Fuzhou, Fujian 350002, China

**Keywords:** A. hypogaea, AhGLK1b, disease resistance, Sclerotinia sclerotiorum, Pseudomonas syringae

## Abstract

GOLDEN2-LIKE (GLK) is a member of the myeloblastosis (MYB) family transcription factor and it plays an important role in the regulation of plastid development and stress tolerance. In this study, a gene named *AhGLK1b* was identified from a cultivated peanut showing down-regulation in response to low calcium with a complete open reading frame (ORF) of 1212 bp. The *AhGLK1b* has 99.26% and 96.28% sequence similarities with its orthologs in *Arachis ipaensis* and *A. duranensis*, respectively. In the peanut, the *AhGLK1b* was localized in the nucleus and demonstrated the highest expression in the leaf, followed by the embryo. Furthermore, the expression of *AhGLK1b* was induced significantly in response to a bacterial pathogen, *Ralstonia solanacearum* infection. Ectopic expression of *AhGLK1b* in *Arabidopsis* showed stronger resistance against important phytopathogenic fungi *S. sclerotiorum*. It also exhibited high resistance to infection of the bacterial pathogen *Pst* DC3000. *AhGLK1b*-expressing *Arabidopsis* induced defense-related genes including PR10 and Phox/Bem 1 (PBI), which are involved in multiple disease resistance. Taken together, the results suggest that *AhGLK1b* might be useful in providing dual resistance to fungal and bacterial pathogens as well as tolerance to abiotic stresses.

## 1. Introduction

Peanut (*Arachis hypogaea*) is an important oilseed and food crop cultivated worldwide. It is a major source of human nutrition, including oil and protein. Being important to both smallholders and large commercial producers, the peanut is widely grown in more than 100 countries every year in tropical and subtropical regions [1]. However, the peanut yield is decreased by a variety of pathogens including fungal, bacterial and viral pathogens. Most of the plant species are vulnerable to a variety of pathogens and pests including fungi, bacteria, oomycetes, nematodes, insects and viruses and in favorable conditions they turn into a disease. Due to preharvest pathogens and pests, an average of 26% crop production was lost worldwide during 2001−2003 [2]. In fighting against these environmental challenges, plants have acquired highly intricate and sophisticated defense mechanisms [3,4]. Several fungal and bacterial pathogens, however, devastate the plant’s defense system, causing disastrous effects on plant growth and decreasing yield.

*Sclerotinia sclerotiorum* is an extremely damaging necrotrophic fungal pathogen of plants. It can infect more than 400 plant species, and caused over $200 million worth of losses in the United States each year [5,6]. Among bacterial pathogens, *Pseudomonas syringae* is one of the model pathogen systems studied in plants. *P. syringae* is identified as a hemi biotrophic pathogen causing diseases in a wide variety of plant hosts including *Arabidopsis* [7,8].

The GOLDEN2-LIKE (GLK) transcription factors (TFs) are members of the glutamic acid/alanine-rich protein (GARP) myeloblastosis (MYB) TFs subfamily [9], which regulate plastid biogenesis by intensifying granule thylakoid stacks and enhance components of nutritional compound [10]. The GLK TF family was first isolated as G2 in maize [11], and was found to be an expression activator of a reporter gene in yeast [12]. GLKs consists of two highly conserved domains: a GCT-box at the C terminal that is specific to GLK genes and a DNA binding domain (DBD) [12]. The hexapeptide sequence AREAEAA at the DBD is extremely conserved among the GARP family [13]. This DBD exists in green algae and land plants, while the GCT-box is found in land plants only.

Most of the well-characterized plant species including *Arabidopsis*, rice, maize, sorghum (*Sorghum bicolor*) and *Physcomitrella* patens, typically contain two GLK genes [12,14,15,16,17,18,19,20,21,22]. *Arabidopsis* (*Arabidopsis thaliana* L.) has two copies of GLK genes, *GLK1* and *GLK2*, which function redundantly to regulate chloroplast development [23]. Tomato (*Solanum lycopersicum* L.) contains two GLKs, *SlGLK1* and *SlGLK2/u*, both expressed in leaves, while *SlGLK2* can be expressed in fruit as well [20,24]. It has been found that these genes encode functionally similar peptides; however, differential expression renders *GLK1* more important in leaves, while *GLK2* is predominant in fruits. A novel ripening-related transcription factor *APRR2-Like* gene that influences pigmentation and ripening in tomato, similar to that of *Arabidopsis* (APRR2) is a gene related to, but different from, the tomato *GLK2* gene [25].

GLKs have been studied extensively in *Arabidopsis* and other species [26,27]. They have a major role in the plant defense system against diseases. The *OsGLK1* gene in rice, play a key role in resistance to pathogen attack [17]. A gain-of-function and loss-of-function study suggested that GLK protein acts upstream of the jasmonate (JA)-dependent signaling pathway in disease susceptibility to *Hyaloperonospora arabidopsidis* (*Hpa Noco2*), and confers strong resistance against necrotrophic fungal pathogens *Botrytis cinerea* via JA-independent plant immunity [27]. Moreover, GLKs confers resistance to viral pathogens. Tobacco (*Nicotiana benthamiana*) GLK gene (*NbGlk1*) interacts with Rx1 and mediates antiviral activity against potato virus X (PVX) [28], and *AtGLK1* has been found to enhance resistance to the cucumber mosaic virus [29].

There are several reports on the GLKs role in plastid regulation, biotic and abiotic stress responses, and resistance to different pathogens. However, response to the important pathogenic fungi *S. sclerotiorum* and virulent bacterial pathogen *Pst* DC3000 has not been reported yet. In the present study a peanut (*A. hypogaea*) gene (named *AhGLK1b*) is found, involved in conferring resistance against fungal as well as bacterial pathogens.

## 2. Materials and Methods 

### 2.1. Plant Materials and Growth Conditions

Seeds of peanut (*A. hypogaea*) cultivar Minhua-6 and *A. thaliana*, ecotype Colombia (Col-0) and tobacco (*Nicotiana benthamiana*) were provided by the Oil Crops Research Institute, Fujian Agriculture and Forestry University. Peanut seeds were sown in plastic pots with normal soil mixed with sand. *Arabidopsis* seeds were first surface sterilized using a standard sterilization method, spread evenly on MS medium (Solarbio, Beijing, China) plates and grown at 22 °C under controlled environmental conditions of 16/8 h light/dark photoperiod with 60% relative humidity and light intensity of 150 µmol photons/m^2^/s. The seedlings were transplanted into plastic pots containing moistened potting soil (Pindstrup substrate, Pindstrup Horticulture Ltd., Shanghai, China) for another 7−8 weeks. Through continuous selfing up to F_4_ generation, pure lines were obtained that were used in functional analysis.

Peanut (Minhua-6) plants were grown in pots under biotic and abiotic stresses in the greenhouse at Fujian Agriculture and Forestry University (FAFU). For calcium stress, peanut plants were grown in the field (summer season, March–July, 2009) at Pingtan, a county in Fujian Province, China. The field contained 0.245 cMol kg^–1^ exchangeable Ca^2+^ content in the soil. This is a low level of Ca^2+^, considered as the Ca^2+^ deficiency treatment. Peanut grown in the same soil, fertilized with 75 kg (667 m^–2^) plaster was used as a control (Ca^2+^ sufficiency treatment). After fertilization, the exchangeable Ca^2+^ content in the soil increased to 1.298 cMol kg^–1^ soil. Normally, less than 1.20 cMol kg^–1^ Ca^2+^ content in soil is the critical value that can result in embryo abortion in peanuts. Embryos (5, 10, 15 and 25 dpp) were collected for RNA extraction. For cold treatment, peanut plants with 4 leaves stage were treated with a low temperature (4 °C) in the growth chamber, while samples at room temperature (28 °C) were taken as a control. Leaf and root samples were taken at 3, 6, 12, 24 and 48 h after treatment for RNA extraction. Under drought stress, irrigation of peanut plants was stopped at the flowering stage with 8 leaves, while plants with regular irrigation were taken as a control, and samples were collected for RNA extraction at 3, 6, 9 and 12 days after treatment. For biotic stress response, resistant (Yueyou-92) and susceptible (Xinhuixiaoli) peanut varieties were inoculated with the bacterial pathogen *R. solanacearum* (*Rs*-P.362200-060707-2-2) at the 7–8 leaf stage, according to previous studies [30,31]. Leaf samples were collected at 0, 3, 6, 12, 24, 48 and 72 h post inoculation (hpi) for RNA isolation. 

### 2.2. Microarray Analysis 

In previous study, a large-scale 454 pyrosequencing was performed for peanut different tissues and responses to biotic- and abiotic-stresses (see Section 2.1) and finally obtained 101,344 unigenes. A high-density gene chip (12 × 135 K probes in a slide) was designed with these unigene probes of 60-base oligonucleotides. Three or six probes for each unigene were devised. In order to identify the calcium deficiency responsive genes in the peanut embryo, total RNAs of embryos under calcium deficient and sufficient conditions were extracted for hybridization. Meanwhile, total RNAs of eight organs and tissues (Root, shoot, leaf, flower, peg, testa, pericarp and embryo) at different growing stages and leaves under various biotic and abiotic-stresses were also extracted to demonstrate the gene expression profile (unpublished data; http://www.peanutgr.fafu.edu.cn/). Microarray hybridization, washing and scanning were done as specified by the NimbleGen guide provided by the Capital Bio Corporation, Beijing, China. Resultant gene expression data were generated by the robust multichip average (RMA) algorithm and normalized by the quantile normalization method (QNM). All hybridizations were analyzed for gene expression intensity and expression levels among the 8 tissues and under various stress conditions. All experiments were performed in three biological replicates. Data were analyzed according to Chen et al. [32]. *AhGLK1b* gene was selected based on its down-regulation for >2 folds under deficiency calcium and higher expression in leaf, embryo and in response to bacterial inoculation.

### 2.3. Full-Length cDNA Cloning

The cDNA sequences of identified target genes were generated by a microarray analysis. The full-length cDNA sequence of *AhGLK1b* was cloned by Gateway technology (Invitrogen, Beijing, China). Total RNA was extracted from young leaves of peanut Minhua-6 using the modified CTAB method and converted to cDNA, as described previously [32]. The 1212 bp coding sequence of peanut (*AhGLK1b*) was amplified from *A. hypogaea* cDNA using AhGLK-20269-F and AhGLK-20269-R primers (Table A1) with attB sites, following thermal cycler conditions: 94 °C for 5 min; 35 cycles at 94 °C for 30 s, 55 °C for 30 s, 72 °C for 1 min 15 s and a final extension of 72 °C for 10 min. The PCR products were sequenced using the Sanger sequencing method, and cloned. Full-length cDNA and DNA sequences of *AhGLK1b* were assembled after sequencing and cloned from reverse transcribed and genomic DNA using a specific set of primers.

### 2.4. Sequence and Phylogenetic Analysis

For biological function determination of *AhGLK1b*, the EMBL-EBI Quick-Go gene ontology tool (www.ebi.ac.uk/QuickGO/GTerm) was used, while molecular mass of protein and theoretical pI value were predicted by Expasy (https://web.expasy.org/compute_pi/). The conserved domains and functional sites were predicted by the pfam tool (http://pfam.xfam.org/search/sequence). Sequence homology of *AhGLK1b* was performed using BLASTX and BLASTN analysis (https://blast.ncbi.nlm.nih.gov/Blast.cgi). For determination of gene location in the genome of peanut, BLASTN was also used in the Peanut Genome Resource databank and PeanutBase (http://peanutgr.fafu.edu.cn/, https://peanutbase.org/). Closely related proteins with high similarity were selected from the BLASTX search. Thirteen proteins were aligned using multiple sequence alignment with Clustal-O (Mview 1.63). A phylogenetic tree was constructed from 20 functionally similar proteins of the Fabaceae family and other related species using Mega 7.0 [33].

The complete ORF of *AhGLK1b* sequence was amplified with LA Taq (Takara, Dalian, China). PCR amplicons were then cloned into pDONOR207 (Invitrogen) plasmid by the BP reaction (Invitrogen, Beijing, China) following the Gateway manufacturer’s instructions. The PCR product of the BP reaction was subsequently transferred to the pK7WG2.0 Gateway vector using LR clonase enzyme (Invitrogen, Beijing, China). The resulting construct containing CaMV35S promotor was designated as pK7WG2.0::*AhGLK1b*-OE. The overexpression vector was transferred into *Agrobacterium tumefaciens* GV3101 strain and verified with PCR amplification followed by sequencing prior to *Arabidopsis* plant transformation.

### 2.5. Subcellular Localization

For subcellular localization, a full-length *AhGLK1b* ORF of 1209 bp fragment (without a stop-codon) was amplified with LA-Taq (Takara, Dalian China) with gene specific primers AhGLK-20269-GFP-F and AhGLK-20269-GFP-R (Table A1). The PCR amplified products were then cloned into pDONR207 (Invitrogen, Beijing, China) and subsequently transformed into pK7FWG2.0 cloning vector, containing over expressing green fluorescent protein (35S::GFP) fusion. The generated construct pK7FWG2.0::*AhGLK1b*-GFP was verified by PCR followed by sequencing, before transformation in the tobacco plant. The construct pK7FWG2.0::*AhGLK1b*-GFP and vector control (35S::GFP) were transformed into *Agrobacterium tumefaciens* strain EHA105, cultured in the induction medium (10 mM ethanesulfonic acid, pH 5.7; 200 mM Acetosyringone; 10 mM MgCl_2_). The cells were harvested by centrifugation, optical density was adjusted to OD_600_ = 0.8, and cells were injected into *N. benthamiana* leaves using a needleless syringe [34]. GFP fluorescence was visualized 2 days post agroinfiltration under fluorescence microscope with a 505–530 nm bandpass emission filter and an excitation wavelength of 488 nm. The GFP florescence was observed and photographed using a laser-scanning confocal fluorescence microscope (Leica TCS SP8, Solms, Germany).

### 2.6. Development of Arabidopsis Transgenic Lines

*Agrobacterium tumefaciens* strain GV3101 harboring pK7WG2.0::*AhGLK1b*-OE cloning vector was grown in liquid culture YEB medium supplemented with 75 µg/mL rifampicin and 50 µg/mL kanamycin at 28 °C overnight. Cells were harvested by centrifugation at 4000 rpm for 10 min at room temperature, and pellet was resuspended in 500 mL infiltration medium (5.0% sucrose, 0.02% Silwet L-77) to a final OD_600_ = 0.8 and transformed in *Arabidopsis* plants using the *Agrobacterium*-mediated floral dip method [35]. Plants were grown for a further 3–5 weeks until maturation. *AhGLK1b*-expressing *Arabidopsis* positive lines were screened by germinating on solid MS medium (Solarbio, Beijing, China) supplemented with 100 µg/mL kanamycin (Sigma). The surviving positive plants were transferred to soil and grown under controlled growth conditions and confirmed by PCR. Positive transformants were advanced to homozygous lines by successive generation on the MS medium with 100 µg/mL kanamycin. Transgenes were confirmed by PCR using 35s-ID forward and reverse primers (Table A1), and further confirmed by sequencing of amplicons. Six transgenic lines were selected and two were used for bioassays.

### 2.7. PCR and Semi-Quantitative Real-Time PCR (sqRT-PCR) Genotyping for Transgene Confirmation

DNA was extracted from *AhGLK1b*-expressing *Arabidopsis* lines using a modified CTAB method [36]. RNA was extracted from leaves using TRIzol^®^ Reagent (Solarbio, Beijing, China) following the manufacturer’s instructions. PCR conditions were as follows: 94 °C for 5 min; 35 cycles at 94 °C for 30 s, 55 °C for 30 s, 72 °C for 1 min and 15 s and a final extension at 72 °C for 10 min. PCR amplicons were visualized on 1% agarose gel.

### 2.8. S. sclerotiorum Bioassays

The fungal pathogen *S. sclerotiorum* isolate 1980 was cultured on PDA medium three days prior to inoculation and kept in the dark at room temperature. Four to five-week-old AhGLK1b and Col-0 plants (2–4 leaves per plant) were inoculated with fresh grown fungal agar plugs about 2 mm in diameter, and kept under humid conditions. Disease development was assessed by necrotic area percentage of individual plant, scored after lesions. Lesion diameter was measured using ImageJ software. At different time points, fungal growth was observed in inoculated leaves. Samples were collected from inoculated leaves at 0, 3, 6, 12 and 24 hpi, transferred immediately into liquid nitrogen and stored at −80 °C for RNA extraction. Total RNA was extracted using TRIzol^®^ reagent, and 1 μg of total RNA was converted into cDNA using a first-strand Prime Script^®^ synthesis kit (Prime Script^®^ RTase reagent, Takara, Dalian, China). Expression analysis was checked by qRT-PCR amplification. Photos were taken at 36 hpi. 

### 2.9. Pst DC3000 Bioassays in Transgenic Arabidopsis

Four-weeks-old transgenic *Arabidopsis* plants AhGLK1b-5 and AhGKL1-6 along with Col-0 leaves were challenged by the bacterial pathogen *Pst* DC3000 grown in King’s B medium (supplemented with 25 μg/mL rifampicin and 100 μg/mL kanamycin) [37]. The cells were harvested by centrifugation at 4000 rpm for 10 min and re-suspended in inoculation medium (10 mM MgCl_2_, 25 μg/mL rifampicin, 0.015% v⁄v Silwet L-77). The optical density was adjusted to OD_600_ = 0.02 and infiltrated into the abaxial side of the leaves using a 1 mL needleless syringe [38]. Two leaves per plant of transgenic lines along with Col-0 were infiltrated with *pst* DC3000. Plants were kept in plastic tray with a transparent plastic cover to maintain optimum humidity. Samples were taken in 2 mL centrifuge tubes at 0, 3, 6, 12, 24 and 48 hpi for RNA extraction. Disease symptoms were observed from time to time and photos were taken 48 h post inoculation.

### 2.10. Quantitative Real-Time PCR (qRT-PCR)

Total RNA was extracted from *AhGLK1b*-expressing and wild-type *Arabidopsis* lines using the TRIzol^®^ Reagent (Solarbio, Beijing, China) following the manufacturer’s instructions. cDNA was prepared by reverse transcription PCR (RT-PCR) using PrimeScript^TM^ RTase (Takara, Dalian, China) following the manufacturer’s instructions. A Master Cycle Rep Realplex (Eppendorf, Hamburg, Germany) was used for qRT-PCR analysis using SYBR Premix Ex II Taq (Perfect Real Time; Takara, Dalian, China). For the relative expression level of target genes, real-time PCR was performed using gene specific primers (Table A1). The amplification mixture was prepared (20 µL), containing SYBR Premix Ex II Taq (2×) 10 µL, 0.2 µL (F/R) PCR primers and 2 µL diluted cDNA. For each gene, three biological replicates were used and the experiment was repeated three times. Thermal cycler conditions were as follows: 95 °C for 5 min; 40 cycles at 95 °C for 5 s, 60 °C for 30 s, 72 °C for 30 s and 95 °C for 15 s, 60 °C for 1 min, 95 for 15 s and 60 °C for 15 s. Amplification specificity was confirmed by melting curve analysis. The relative transcription level of target and control genes was calculated as ∆∆CT^–2^ [39] (∆∆Ct = (CT_gene_ − CT_actin_)_treat_ − (CT_gene_ − CT_actin_)_control_). As internal reference, *Ahactin* was used for detection of the relative expression level of *AhGLK1b* in the peanut. *AtUBC21* (AT5G25760)2 [40,41] or *Ahactin* was used as an internal reference for detection of the relative expression level of *AhGLK1b* under different treatments in *Arabidopsis*. 

### 2.11. Statistical Analysis

Gene expression data of microarray analysis were analyzed by one-way analysis of variance, and the differences among means were evaluated using a Tukey post hoc multiple comparison test (IBM SPSS Statistic 22). Statistically significant differences in the figures were defined as those with a *p* value < 0.05. Expression data were analyzed using IBM Statistics 22 for significant differences from their control. *p* values were analyzed by Student’s *t* test. Significance level is indicated as **p value < 0.05 and ***p value < 0.01. Measuring necrosis, ImageJ 1.51K was used and their percentage was calculated by Microsoft Excel.

## 3. Results

### 3.1. Cloning and Sequence Analysis of AhGLK1b

**Figure 1:**
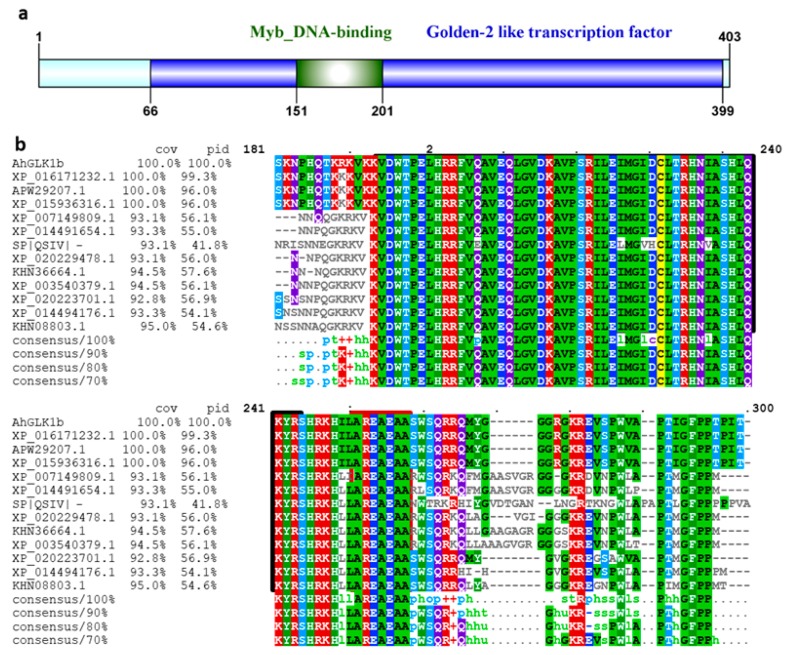
Multiple sequence alignment of *AhGLK1b* in the Fabaceae family and related species, sharing sequence homology. (**a**) Graphical representation of the *AhGLK1b* conserved domain, the sketch was drawn with IBS 1.0.3. Myb_DNA-Binding domain and Golden-2 like transcription factor are the major domains. (**b**) Clustal-O (Mview 1.63) multiple sequence alignment of AhGLK1b proteins. Black box indicates conserved Myb-DNA Binding Domain. While the red box depicts a hexapeptide one of the most conserved sequences. *AhGLK1b* shares conserved domain with other members of the Leguminosae family; XP_016171232.1 (*Arachis ipaensis*), APW29207.1 (*Arachis hypogaea*), XP_015936316.1 (*Arachis duranensis*), SP|QSIV| (*Arabidopsis thaliana*), XP_020229478.1 (*Cajanus cajan*), XP_007149809.1 (*Phaseolus vulgaris*), KHN36664.1 (*Glycine soja*), XP_003540379.1 (*Glycine max*), XP_014494176.1 (*Vigna radiata*), XP_020223701.1 (*Cajanus cajan*), XP_014491654.1 (*Vigna radiata*) and KHN08803.1 (*Glycine soja*).

The *GLK1b,* isolated from embryo by microarray analysis, was identified with an open reading frame of 1212 bp by homology BLAST in NCBI, exhibiting high similarity with the GLK gene family. A 1212 bp of full-length cDNA sequence of *GLK1* was cloned from a cultivated peanut by RT-PCR using specific primers. The CDS sequence was deposited to GenBank under accession number MK952147 and designated as *AhGLK1b* (*A. hypogaea GOLDEN2-like 1 transcription factor*). Sequence analysis revealed that the deduced *AhGLK1b* encoded 403 amino acid polypeptides with a molecular mass of 44.07 kDa and a theoretical pI of 8.72. Multiple sequence alignment of AhGLK1b shared a conserved Myb-DNA binding domain of 50 amino acids at 151−200 across the Fabaceae family (Figure 1). The Myb-DNA binding domain is associated with chloroplast-related genes and functions in the disease defense system in plants. The sequence shared close homology (70%–99%) with GLKs of Fabaceae and related species. AhGLK1b protein shared 52% sequence identity with transcription activator *AtGLK1*, which is a disease defense related protein, conferring resistance to the fungal pathogen *Fusarium graminearum* [42].

### 3.2. Phylogenetic Analysis

**Figure 2:**
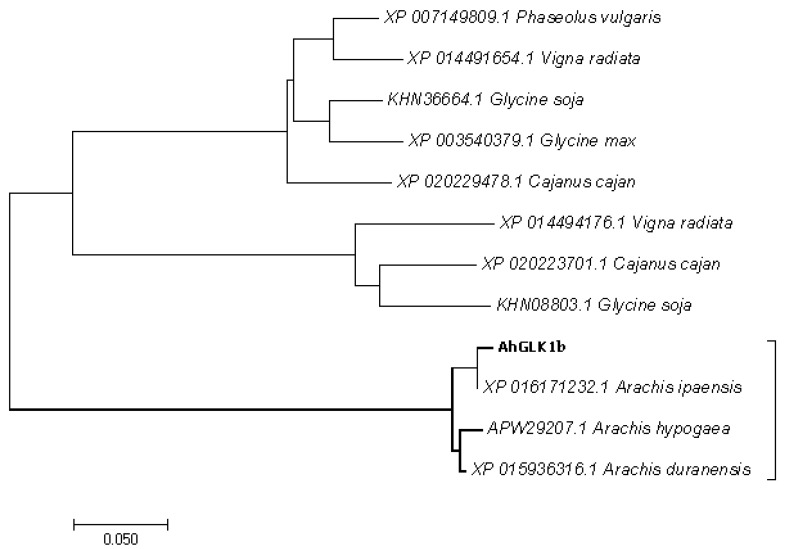
Phylogenetic tree analysis of *AhGLK1b* with GLKs of legumes, sharing the same group with those from *Arachis ipaensis*, *Arachis hypogaea* and *Arachis duranensis*. Alignment was performed in ClustalW2 and phylogenetic tree was generated by the neighbor-joining algorithm in Mega 7.0. Bootstrap values (1000 replicates) are shown in percentages at the branch nodes.

To elaborate the relationship with other genes, the evolutionary history of *AhGLK1b* along with 20 other GLKs from legumes and related species was inferred by phylogenetic analysis using the neighbor-joining method with a bootstrap test (1000 replicates). A phylogenetic tree was constructed with MEGA7 software (Figure 2). *AhGLK1b* is basically known for chloroplast biogenesis and photosynthesis-related genes. However, *AhGLK1b* was also found to be related to disease resistant genes. This gene has a close relationship with *Arabidopsis* GLK, which has a distinct role in plant disease resistance. The homology indicates that GLKs may play a key role in the plant defense system against pathogens.

### 3.3. Localization of AhGLK1b in the Nucleus

**Figure 3:**
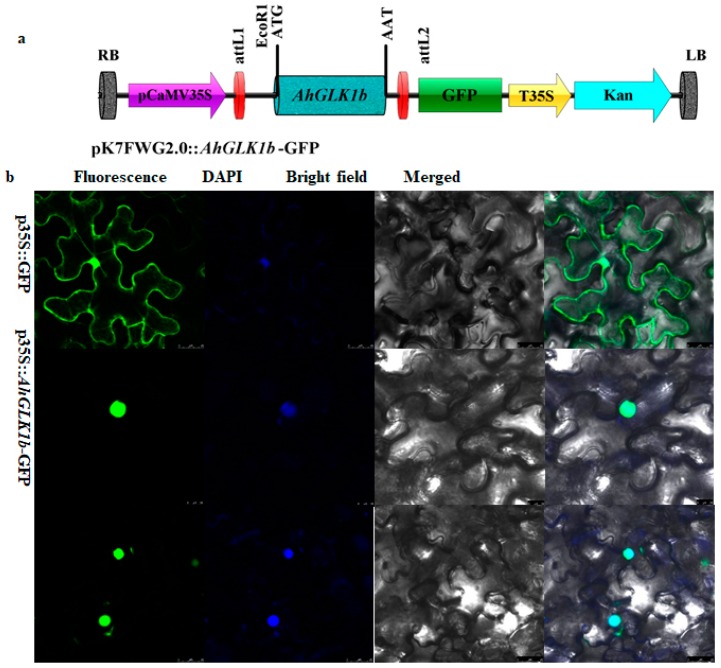
Nuclear localization of *AhGLK1b* in tobacco leaves by transient expression using the agroinfiltration method. (**a**) Schematic diagram of the green fluorescent protein (GFP) vector with the p35S promoter. (**b**) Localization of *AhGLK1b* in the nucleus. The fluorescence signal was detected in epidermal cells using a confocal microscope, nucleus was stained with DAPI. The empty-vector pK7FWG2.0 was used as a control. pK7FWG2.0::*AhGLK1b*-GFP expressing translationally fused *AhGLK1b*-GFP and cells were visualized at different planes. Scale bar = 638 µm.

AhGLK1b predicted that was localized in the nucleus by a subcellular localization tool (http://abi.inf.uni-tuebingen.de/Services/YLoc/webloc.cgi). For confirmation, we constructed *AhGLK1b*-green fluorescent protein (GFP) fusion, driven by a CAMV35S constitutive promoter (35S::GFP). The *AhGLK1b::GFP* fusion gene was transformed into the *Agrobacterium tumefaciens* strain EHA-105, which was further infiltrated into tobacco *N. benthamiana* leaves using a needleless syringe. Observations confirmed exclusive localization of *AhGLK1b* in the nucleus while the GFP control vector appeared in multiple subcellular sections, including the nucleus, cytoplasm and cell membrane (Figure 3). The results confirmed that *AhGLK1b*, a transcription factor, was localized and functioned in the nucleus.

### 3.4. Expression Pattern of AhGLK1b in Different Peanut Tissues

**Figure 4:**
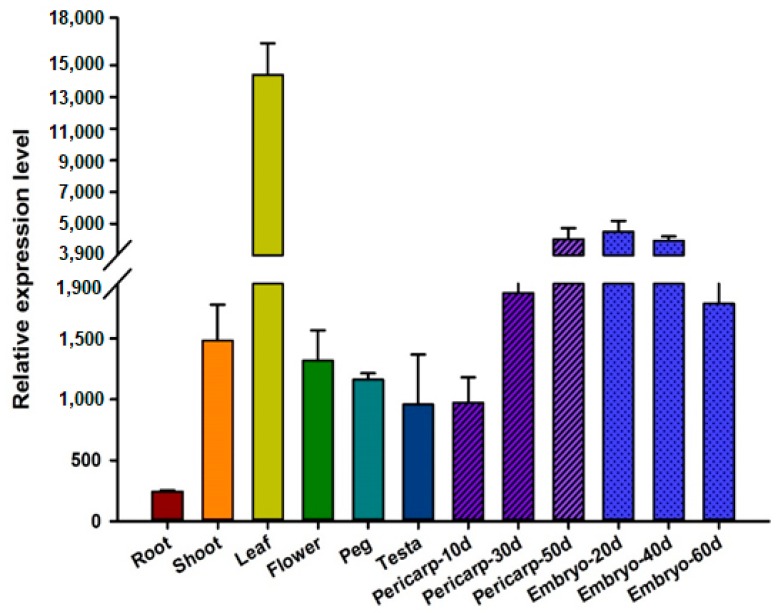
Microarray analysis and expression pattern of *AhGLK1b* in peanut (Minhua-6) tissues and in response to plant growth regulators. *AhGLK1b* expression in different tissues and developing pericarp and embryo in the peanut (d represent days after pegging).

By microarray analysis with a density of nearly 100,000 unigenes in chips, *AhGLK1b* expressions were characterized. For better comparisons, nonamplified double-strand cDNA was used for microarray hybridization. The *AhGLK1b* showed a tissue-specific expression pattern among organs or tissues; the highest expression level was observed in the leaf with several folds over other tissues, followed by embryo, pericarp, shoots and flower (Figure 4). The lowest expression level was noted in roots.

### 3.5. AhGLK1b Response to Biotic and Abiotic Stresses

**Figure 5:**
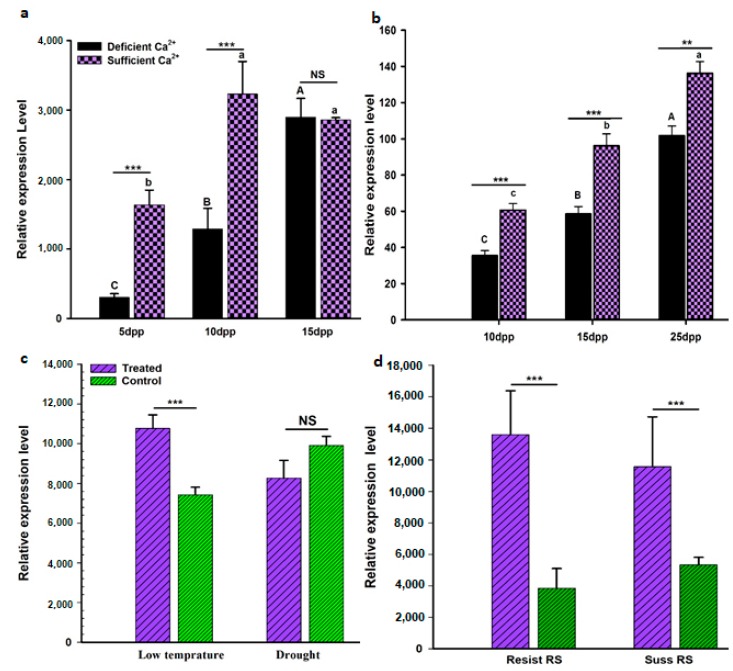
Microarray or qRT-PCR analysis of *AhGLK1b* transcription levels responding to different abiotic and biotic stresses. (**a**) Microarray analysis of *AhGLK1b* expression in response to deficient and sufficient Ca^2+^ in a peanut developing embryo. (**b**) qRT-PCR analysis of *AhGLK1b* in a peanut embryo at different developmental stages under Ca^2+^ stresses. (**c**) Microarray analysis of *AhGLK1b* expression in peanut seedlings in response to low temperature and drought stresses. (**d**) qRT-PCR analysis of relative expressions of *AhGLK1b* in resistant and susceptible peanut cultivars responding to the *R. solanacearum* challenge. The samples pods or leaves were collected from more than thirty plants or seedlings, with 10–15 plants or more than 40 pods mixed as one biological repeat. Data represent means of three biological replicates ± SD, asterisks indicate statistical significance in comparison with the control (Student’s *t*-test, significance levels of **p < 0.05, ***p < 0.01).

Microarray analysis was performed to study the *AhGLK1b* response to biotic and abiotic stresses. Peanut variety Minhua-6 subjected to abiotic stresses (deficient Ca^2+^, cold and drought) and biotic stresses by inoculation of the bacterial pathogen *R. solanacearum* (bacterial wilt causative agent). The plants challenged with deficient Ca^2+^ stress in soil demonstrated a significant decrease of *AhGLK1b* expressions in embryos at 10, 15 and 25 DAP (days after pegging), using nonamplified double strand cDNA for hybridizations (Figure 5a). For further confirmation, embryo samples taken at 10, 15 and 25 DAP assayed by qRT-PCR showed increased expression with embryo development (Figure 5b) and downregulated expression upon deficient Ca^2+^ stress. The results indicate that the gene has a function in embryo development. In addition, *AhGLK1b* was also upregulated in response to low temperature and drought (Figure 5c). Resistant and susceptible peanut varieties (Yueyou-92 and Xinhuexiaoli, respectively) inoculated with *R. solanacearum* presented upregulation very significantly in peanut, which was also supported in a number of other plant species [30,43,44]. All the results may indicate the *AhGLK1b* transcription factor plays a role in defensive or tolerant responses to biotic and abiotic stresses.

### 3.6. Overexpression of AhGLK1b in Arabidopsis Enhances Resistance to Fungal Pathogen S. sclerotiorum 

**Figure 6:**
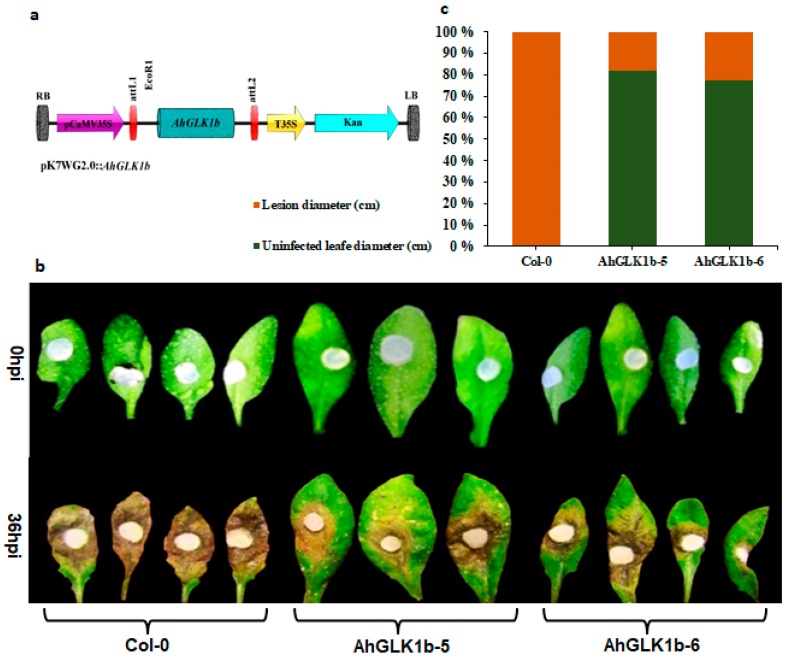
Antifungal assay of *AhGLK1b*-overexpressing *Arabidopsis* lines. (**a**) Schematic diagram of overexpression vector with the p35S promoter. (**b**) AhGLK1b-5 and AhGLK1b-6 along with Col-0. *Arabidopsis* lines were inoculated with equal size (about 2 mm in diameter) of *S. sclerotiorum* agar plug. Forty plants were infected by fungus for each line. Photographs were taken 36 hpi. Note severe chlorosis on Col-0, almost all leaf is decayed while AhGLK1b-5 and AhGLK1b-6 lines has less chlorosis indicating stronger resistance to a fungal pathogen. (**c**) Percent decaying lesion size, measured and calculated using ImageJ software.

**Figure A1:**
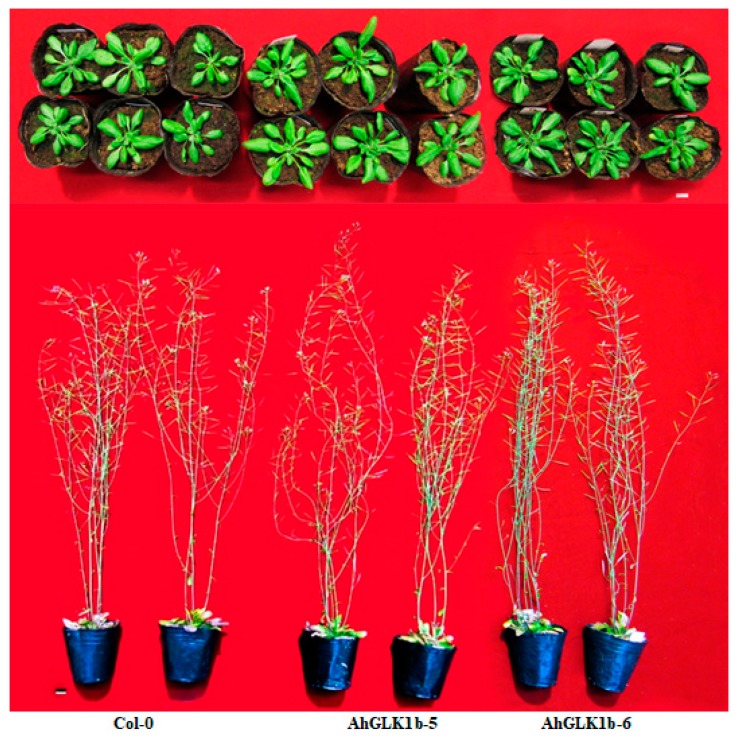
Phenotypic comparison of transgenic Arabidopsis AhGLK1b and wild-type Col-0. Plants before bolting stage and mature plants before dehiscence. AhGLK1b-5 lines were comparatively smaller while AhGLK1b-6 and Col-0 has no significant difference. All plants were grown at same environmental conditions. Scale bar represent 1cm.

**Figure A2:**
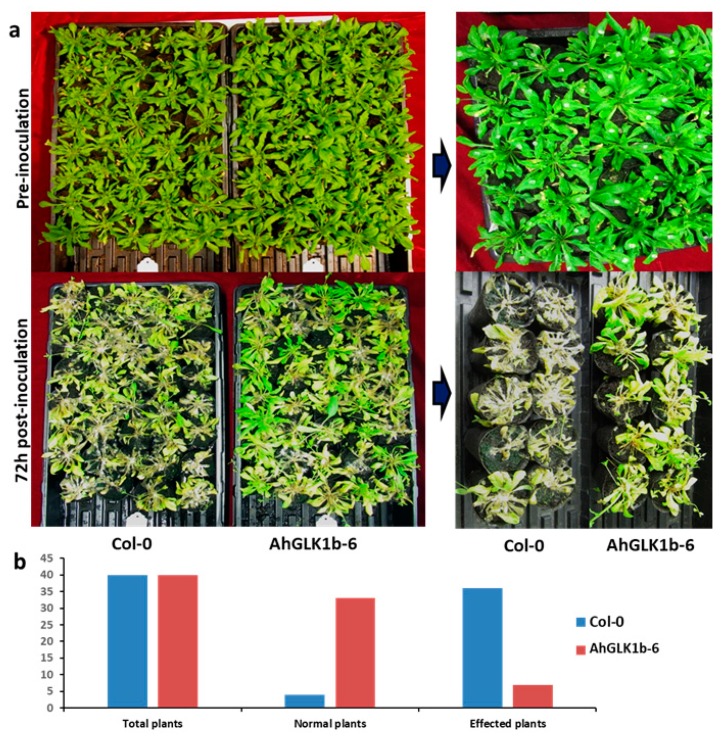
Response of transgenic (AhGLK1b-6) and non-transgenic (Col-0) plants to fungal pathogen *S. sclerotiorum*. For each line 40 plants were inoculated with an equal volume of agar plaque containing *S. sclerotiorum*.

Transgenic *Arabidopsis* lines expressing *AhGLK1b* were phenotypically observed throughout their growing stages. Two transgenic lines named AhGLK1b-5 and AhGLK1b-6 were selected for further experiments. No significant difference in phenotype was observed under normal growth conditions (Figure A1). Forty pure transgenic (T_4_) plants for each line were evaluated for disease resistance by challenging them with the fungal pathogen *S. sclerotiorum* strain 1980. In comparison with wild-type (Col-0), *AhGLK1b* lines showed a stronger resistance against *S. sclerotiorum* and restricted fungal growth significantly (Figure 6). Disease symptoms were clearly visible in both transgenic and control plants 36 h post inoculation. Almost total (99%) leaf of Col-0 was decayed by fungal invasion while AhGLK1b-5 and AhGLK1b-6 leaves showed less effect (20%–25%). Complete plants survival rate was also observed 72 hpi (hours post inoculation). Control (Col-0) plants showed less (10%) survival rate while transgenic plants (AhGLK1b-6) showed strong resistance with a higher (82.5%) survival rate (Figure A2). 

### 3.7. AhGLK1b Confers Tolerance to Bacterial Pathogen Pst DC3000

**Figure 7:**
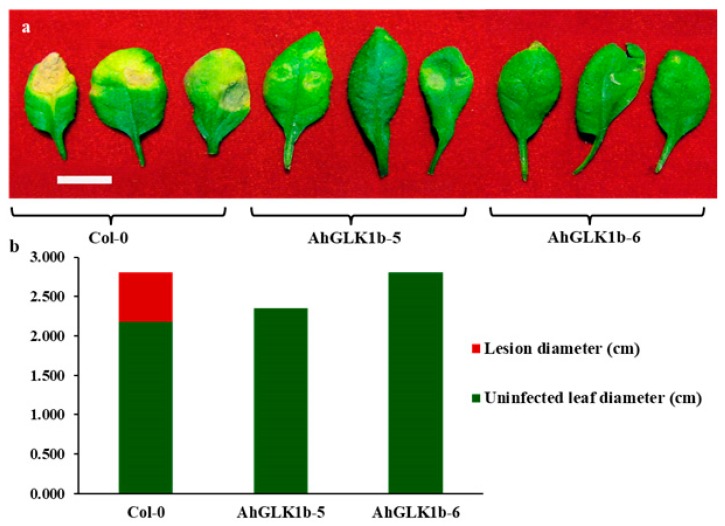
*AhGLK1b*-overexpressing *Arabidopsis* plants are tolerant to pathogenic bacteria, *Pst* DC3000. (**a**) AhGLK1b-5 and AhGLK1b-6 *Arabidopsis* lines conferring resistance against *Pst* DC3000. Forty plants were infected by *Pst* DC3000 for each line. Disease symptoms caused by *Pst* DC3000 were syringe infiltrated with OD_600_ = 0.02. Leaves were photographed 48-hours after inoculation. (**b**) Measurement of lesion diameter in AhGLK1b and non-transgenic *Arabidopsis* (Col-0) leaves. Scale bar represents 1 cm.

To elucidate the disease tolerance of overexpressed *AhGLK1b*, 40 plants each of transgenic and nontransgenic *Arabidopsis* lines were inoculated with the bacterial pathogen *Pst* DC3000. Col-0 plants were affected by *Pst* DC3000, while AhGLK1b-5 and AhGLK1b-6 lines showed strong resistance and no disease symptoms (Figure 7). The results indicate that *AhGLK1b*-overexpressing *Arabidopsis* lines have the capability of strong resistance against *Pst* DC3000 and may enhance the plant disease defense system.

### 3.8. Defense-Related Genes Response to Overexpression of AhGLK1b

**Figure 8:**
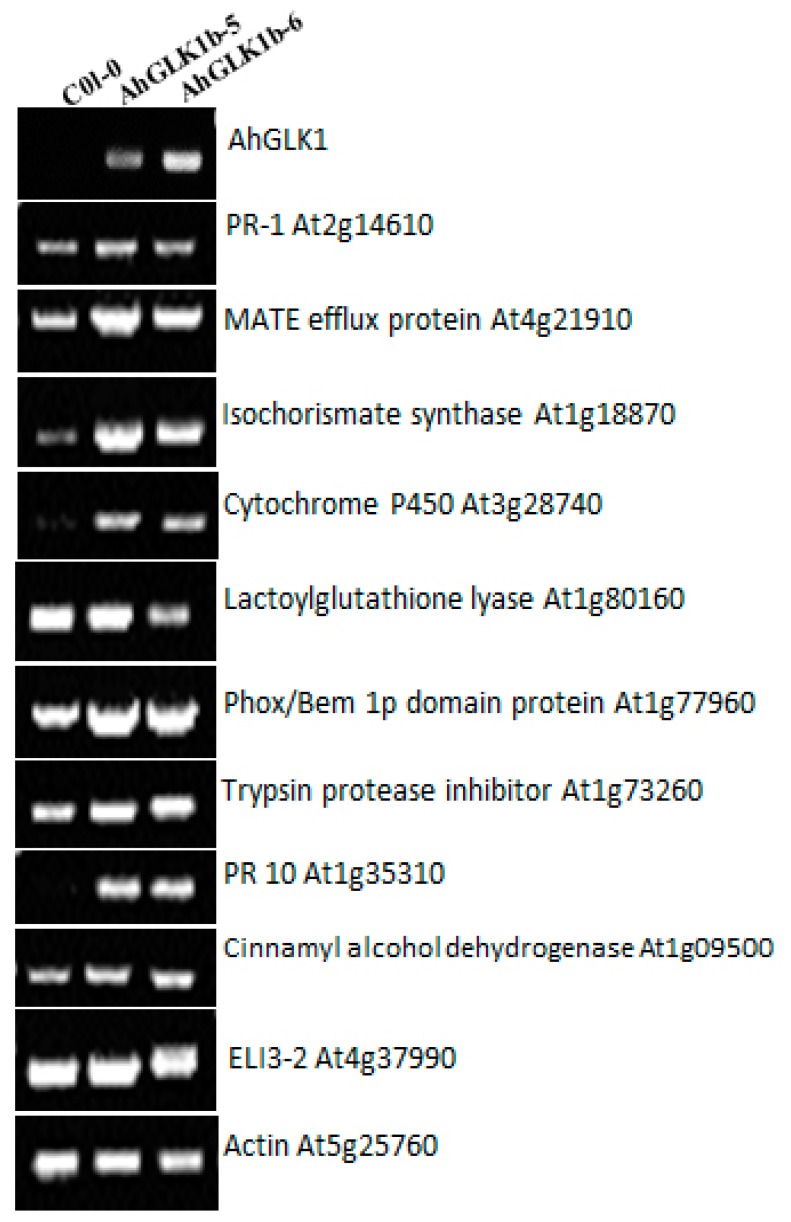
RT-PCR analysis of defense related genes in ectopically expressed *AhGLK1b* in *Arabidopsis*. Lanes Ck depicts Col-0, A represents AhGLK1b-5 while B indicates AhGLK1b-6 lines.

To determine the function of *AhGLK1b* in the plant defense system against pathogens, the expression of key *Arabidopsis* defense-related genes were measured in transgenic and control leaves (Figure 8) in a variant pattern. As *AhGLK1b* was cloned from a peanut, Col-0 did not show transcripts. *Arabidopsis* PR10 protein was upregulated and highly expressed, while PR1 was slightly upregulated. Among other defense marker genes, isochorismate synthase, multidrug and toxin extrusion (MATE) efflux protein, cytochrome P450, Phox/Bem 1p domain protein and trypsin protease inhibitor were all upregulated expression in AhGLK1b transgenic lines. Moreover, cinnamyl alcohol dehydrogenase was upregulated, while ELI3-2, lactoylglutathione lyase and *AtGLK1* showed similar expression with nontransgenic control Col-0 lines.

## 4. Discussion

### 4.1. AhGLK1bAhGLK1b Conferes Resistance to Biotic and Abiotic Stresses

**Figure A3:**
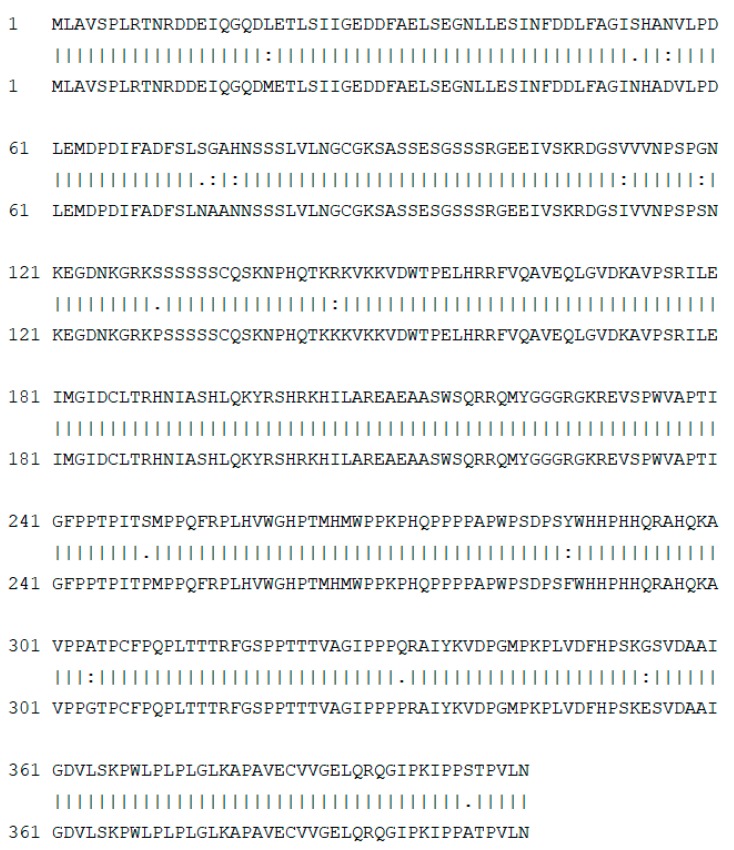
Pairwise alignment of cloned AhGLK1b Minhua-6 (B sub-genome) and AhGLK1_Yueyou-7 (A sub-genome). Upper line: AhGLK1b_Minhua-6, from 1 to 403, Lower line: AhGLK1_Yueyou-7, from 1 to 403, identity= 96.03% (387/403) gap=0.00%(0/403). Identical nucleotide residues are marked as | while conserved and semi-conserved substitutions are depicted as : and ., respectively.

GLKs have been studied in various plant species and extensively in *Arabidopsis*. Overexpression of GLK intensifies fruit photosynthesis-gene expression and chloroplast development, enriching carotenoids and carbohydrates in ripe fruit [28]. In the present study, a 1212 bp fragment encoding 403 amino acid proteins was identified from cultivated peanut var. Minhua-6. The deduced 403 amino acids share a close sequence homology with GLK1 of the Myb-GARP transcription factor family, designated as *AhGLK1bAhGLK1b*. Members of this family directly bind to the promoters of their target genes and perform as transcriptional regulators [22]. Phylogenetic analysis showed that *AhGLK1b* is closely related to GLK1 of wild peanuts *A. ipaensis* and *A. duranensis*. Moreover, *AhGLK1b* shares 96% similarity with a recently studied peanut GLK1, *AhGLK1* [45] (Figure A3). However, our gene *AhGLK1b* is located on the B subgenome of a cultivated peanut, while the recently studied one has a locus at the A subgenome. Gene ontology and GFP analysis showed that *AhGLK1bAhGLK2* was localized in the nucleus (Figure 3). GLK1 transcription factor has been found to confer tolerant to various abiotic stresses, such as ozone [46], salt and drought [45,47]. Our results are consistent in terms of tolerance to cold stress; under low temperature (4 °C), *AhGLK1b* showed a high transcript level. However, there was no significant difference between drought-treated and normal peanut plants. The differences in drought response may be due to the *AhGLK1b* in this study being different from the one cited in [45], in that our gene is located on the B subgenome instead of A subgenome [45]. *AhGLK1b* is clearly regulated under low Ca^2+^ stress; a lower transcript level and downregulation were noted under deficient Ca^2+^ (Figure 5a,b [48]). These results suggest that *AhGLK1b* is a transcription factor that regulates many genes related to biotic [26,27] and abiotic [49,50] stresses and regulates chloroplast development [14,15,22].

### 4.2. Defense-Related Gene Responded to AhGLK1b in Arabidopsis

To elucidate the function of *AhGLK1b* in plant disease resistance, key defense-related genes [26] evaluated in *AhGLK1b*-expressing *Arabidopsis* and control plants were investigated. Marker defense genes include MATE efflux protein (At4g21910), which is a family of multidrug and toxin extrusion efflux transporters essential for disease resistance [51,52]. MATE efflux protein is upregulated in *AhGLK1b*-expressing *Arabidopsis* lines in comparison with Col-0. The results are consistent in terms of resistance to the fungal pathogen *F. graminearum*, which uses the major virulence factor mycotoxin deoxynivalenol (DON) [53,54]. Another highly accumulating gene, PR10, has been found to be upregulated in response to pathogens [55,56] and acquire steroid binding [57] and antimicrobial activities [58]. Other upregulated transcripts encoding the Phox/Bem 1 (PBI) domain protein are related to protein–protein interactions and associated with the activation of NADPH oxidase against pathogens, reactive oxygen system, reactive oxygen species (ROS) and suppression of cell death [59,60,61]. In other defense-associated genes, ELI3-2, an aromatic alcohol dehydrogenase and cinnamyl alcohol dehydrogenase (CAD) [62,63], produced a low transcript level. The results contradict those of Savith et al. [42], possibly because of difference in plant species. Among other defense-related genes, *Arabidopsis* isochorismate synthase (ICSI), a key enzyme in salicylic acid (SA) biosynthesis in chloroplast [64], was upregulated. The upregulation of these marker defense genes in *AhGLK1b-OE Arabidopsis* indicates the role of *AhGLK1b* mediated resistance in the plant disease defense system. 

### 4.3. AhGLK1b Enhace Resistance to Fungal Pathogen S. sclerotiorum

*AtGLK1* (*35S:AtGLK1*) overexpression induced resistance to the cereal fungal pathogen *F. graminearum* in *Arabidopsis* [26,65,66]. GLK was reported to act upstream of JA signaling in disease susceptibility to *Hpa Noco2* [27]. Constitutive overexpression of *AtGLK1* also confers high resistance to the pathogenic fungus *Botrytis cinerea* in JA-independent resistance [27]. *AhGLK1b* has 52% sequence similarity with disease defense-related protein *AtGLK1* transcription activator from *Arabidopsis*, which confer resistance to the fungal pathogen *F. graminearum* [42]. *AhGLK1b*-expressing *Arabidopsis* lines are highly resistant to *S. sclerotiorum*, a model [67] fungal pathogen in *Arabidopsis*. In the current study, we observed that *AhGLK1b* ectopic expression in *Arabidopsis* conferred resistance to *S. sclerotiorum* (Figure 6, A2). *AhGLK1b* showed an enhanced tolerance to *S. sclerotiorum* inoculation and had fewer decaying symptoms compared to control with higher survival rate after 72 hpi with fungal pathogen (Figure A2). *AhGLK1b* might play an important role in the plant defense system against *S. sclerotiorum*. As suggested for other GLK1s, the antifungal activity of *AhGLK1b* might be attributed to reprogrammed gene expression networks, to induce high constitutive expression of genes encoding proteins involved in basal defenses [42], which was consistent with our study as above. 

### 4.4. AhGLK1b Confer Resistance to Bacterial Pathogen Pst DC3000

Induced accumulation of *AhGLK1b* transcript in peanut leaves was noted after *R. solanacearum* inoculation. In both resistant and susceptible peanut cultivars, *AhGLK1b* was upregulated, revealing its function in plant defense against bacterial pathogens. To elucidate these results, we challenged ectopically expressed *AhGLK1b Arabidopsis* plants, with the hemibiotrophic bacterial pathogen *Pst* DC3000 (Figure 7). *AhGLK1b*-expressing *Arabidopsis* lines showed strong resistance in comparison with Col-0. Few studies have reported the function of GLK1 in response to bacterial pathogens. Wang et al. [68] reported downregulation of *AtGLK1* in response to *Pst* DC3000, however, we studied *AhGLK1b* that increased resistance to the pathogen. The possible contrast is the species and sequence differences. Our results indicated that *AhGLK1b* was upregulated after inoculation. Overall results indicate that *AhGLK1b* also confer resistance to *Pst* DC3000 and has a potential role in the plant defense system against bacterial pathogens.

### 4.5. AhGLK1b May be Involved in Embryo Development

*AhGLK1b* was isolated as a down regulated gene responding to deficiency calcium associated with low Ca^2+^ induced embryo abortion. Spatial and temporal characterization of transcripts indicated significant difference. The maximum transcript level was noted in leaves, followed by the embryo, pericarp and stem, while the lowest expression level was observed in roots, signifying its close relation to leaves or embryo development [69]. High expression in leaves and embryos indicates a putative role of *AhGLK1b* in photosynthesis via regulating of chloroplast or plastids [69]. GLK is associated with differentiation of the photosynthetic cell type of maize leaf [69]. These results are consistent with those of previous studies. In various plant species GLK genes regulate chloroplast development [14]. GLK proteins regulate photosynthesis based on cells differential requirements within the leaf [21]. Overexpression of GLK1 and GLK2 induces enhanced expression of chlorophyll related genes, with ectopic chlorophyll accumulation in non-photosynthetic organs [17,21,24]. As a transcription activator of photosynthesis-related genes [10,24,45,70,71,72,73], an increasing transcript level was recorded in developing pericarp and embryo. The maximum transcript level was observed at 50 DAP in pericarp, while embryo showed the highest expression level at 20 DAP. A similar expression pattern was observed in developing embryo under Ca^2+^ stress, elucidating a potential role of *AhGLK1b* in embryo development. GLK genes assist coregulation and synchronization of nuclear photosynthetic genes expression, consequently optimize photosynthetic capacity in various environmental and growth conditions [22]. GLK overexpression boost fruit photosynthesis gene expression and chloroplast development resulting in increased sugar contents and carotenoids in ripe fruit [24]. Taken together, these results suggest that *AhGLK1bb* expression is organ-preferential and time-specific under stressed and unstressed conditions.

## 5. Conclusions

In this study, a calcium deficiency-induced gene *AhGLK1b* identified and characterized with a CDS length of 1212 bp encoding 403 amino acid *AhGLK1b*. This *AhGLK1b* is the GARP-MYB transcription family members, responsible for several functions in the plants. The functional characterization revealed that *AhGLK1b* was responsive to multiple biotic and abiotic stresses. *AhGLK1b* also conferred dual resistance to fungal and bacterial pathogens, by hampering their growth in the host plant. *AhGLK1b* also had the potential to increase tolerance to abiotic stresses in peanut and transgenic *Arabidopsis*. Moreover, the results suggest that *AhGLK1b* might be a suitable candidate to provide tolerance in peanut to multiple stresses.

## Appendix D

**Table A1:** List of primers used in this study.

S. No.	Primer	F/R	Primer sequences (5′to 3′)
			**Cloning (ORF)**
1	AhGLK-20269	F	GGGGACAAGTTTGTACAAAAAAGCAGGCTTCACCATGCTTGCGGTGTCACCTTTG
		R	GGGGACCACTTTGTACAAGAAAGCTGGGTCTTAATTAAGCACAGGAGTTG
2	AhGLK-20269-GFP	F	GGGGACAAGTTTGTACAAAAAAGCAGGCTTCACCATGCTTGCGGTGTCACCTTTG
		R	GGGGACCACTTTGTACAAGAAAGCTGGGTCATTAAGCACAGGAGTTGAG
			Transgenes ID (P35s)
3	AhGLK-trans ID-35s	F	TGATGTGATATCTCCACTGACGTAAG
		R	GATGGATTCACTACGATGGATCCATC
			**Semi-quantitative RT-PCR**
4	qRT-AhGLK	F	TCCCTAGTCTTGAATGGTTG
		R	TGATTGACATGATGAGGAGG
5	AtUBC21 (AT5G25760)1	F	GGCATCAAGAGCGCGACTGT
		R	GGCGAGGCGTGTATACATTT
6	At1g09500 Cinnamyl alcohol dehydrogenase	F	CAACCACATGCACACTAATTC
		R	CCATTGGCTGAAGGAGTCTCG
7	At1g35310 PR10	F	CAACATGCCTCCAAAGCCACTC
		R	ACGCATACAATAACTCTCCCACAC
8	At1g73260 Trypsin protease inhibitor	F	CTATCAAGCCGCCTCACCTA
		R	CTCACCGACCCGCCAGTA
9	At1g77960 Phox/Bem 1p domain protein	F	ATCGCCGTTGTTGAGTCCTTTATG
		R	AGTATTTGTGCCCCTTTGTTCAGC
10	At1g80160 Lactoylglutathione lyase	F	GATGGCGCGTGGTTGTTT
		R	GGGGAGGCTATCGCAGTTG
11	At3g28740 Cytochrome P450	F	CGGCCGCGTAAACTAAACCTA
		R	TCCCGGCAAGTATCATAACAAGTA
12	At4g37990 ELI3-2	F	CGCCGCACCGCTCCTCTG
		R	GCGGTACCTAACGTCGGCTTTCTC
13	At1g18870 Isochorismate synthase	F	AGTGCCCCAGGTCGAGTTTGATG
		R	TCCCGCCTTTCTTTAGTTGATTGG
14	At4g21910 MATE efflux protein	F	GTTGGCACTACCGGCGATACTTA
		R	ACACCCAACCGGAATACCAACAAC
15	At2g14610 PR-1	F	CTCAAGATAGCCCACAAGA
		R	AAGGCCACATATTTTACAT
			**Quantitative RT-PCR**
16	qRT-AhGLK	F	TCCCTAGTCTTGAATGGTTG
		R	TGATTGACATGATGAGGAGG
17	AtUBC21 (AT5G25760)2	F	CTGAGCCGGACAGTCCTCTT
		R	TAGCGGCGAGGCGTGTATAC
